# Regulatory T Cells in Transplantation

**DOI:** 10.1016/j.transproceed.2011.06.050

**Published:** 2011-07

**Authors:** K.J. Wood

**Affiliations:** Transplantation Research Immunology Group, Nuffield Department of Surgical Sciences, University of Oxford, John Radcliffe Hospital, Oxford OX3 9DU, United Kingdom

## Abstract

T Regulatory cells (Treg) play an important role in the induction and maintenance of immunological tolerance to self and alloantigens. Recent findings in experimental transplant models have demonstrated that Treg can control acute and delayed allograft rejection. Preclinical attempts to use Treg as a cellular therapy have been successfully undertaken demonstrating the safety and feasibility of such treatment, suggesting that they have therapeutic potential.

Controlling the immune response to the allograft while retaining the ability to combat infection and prevent tumor formation presents major challenges. Immunosuppressive drugs are clearly very efficient at preventing acute rejection. However, current immunosuppressive therapies are much less effective at allowing the immune system to continue to respond to antigens other than donor alloantigens after transplantation, as they affect the function of all responding T cells irrespective of their antigen specificity.

CD25^+^CD4^+^FOXP3^+^ regulatory T cells are one of the key populations responsible for controlling immune responses to alloantigens and preventing rejection in vivo. Thymus derived or naturally occurring, CD25^+^CD4^+^FOXP3^+^ regulatory T cells are generated as a distinct population during T cell development in the thymus.^1–4^ Importantly for transplantation, CD25^+^CD4^+^FOXP3^+^ regulatory T cells that are phenotypically and functionally similar to the thymus derived population can also be generated or induced after alloantigen exposure both in vivo^5–10^ and ex vivo (for example ^11–14^). We have shown, firstly, that regulatory T cells responsive to alloantigen can arise by both expansion of thymus derived or naturally occurring regulatory T cells and conversion following exposure to alloantigen, and secondly, that T cells in the periphery that are uncommitted to any lineage can be induced to acquire regulatory function, emphasizing the plasticity of T cell differentiation following activation.^15^ In each of these populations of regulatory T cells, sustained expression of FOXP3 is essential for maintaining the transcriptional programme established during their commitment to become a regulatory T cell.^16^

In a transplant recipient, donor alloantigen–reactive regulatory T cells may develop and their activity be sustained in a number of different ways. Although alloantigen pretreatment can induce and/or expand alloantigen reactive regulatory T cells, as discussed above,^5,8^ arguably a more important source of antigen is the graft itself, as the immune system of a transplant recipient is constantly exposed to donor alloantigens while the graft continues to function. In a mouse model, we demonstrated that the presence of the allograft as the source of donor alloantigen was essential for maintaining the unresponsive state.^17^ This source of antigen is unique to transplantation and may be important in enabling FOXP3 expression to be stabilized and sustained and/or for regulatory T cells populations to be renewed in vivo throughout the posttransplant course,^18^ thereby allowing rejection to be controlled.

Alloantigen induced regulatory T cells can prevent acute, as well as delayed or chronic graft rejection. In mouse models, our own laboratory has demonstrated that regulatory T cells induced in response to alloantigen in vivo can prevent the rejection of heart allografts in naïve mice with an intact immune repertoire,^5,19^ as well as of heart, skin, and islet allografts in more subtle adoptive transfer models using immunodificient hosts that enable the mechanisms of suppression to be investigated.^6,8,13^ Moreover, these same populations of alloantigen reactive regulatory T cells can prevent the development of transplant arteriosclerosis in mouse and humanized mouse models,^20–23^ a feature of allograft rejection that is especially hard to control by experimental tolerance induction strategies^24^ and immunosuppressive drug therapy.

The specificity of alloantigen reactive regulatory T cells generated following antigen exposure has been investigated in a number of different experimental systems. Our own data suggest that in vivo, in mice with long-term surviving allografts, regulatory T cells respond to alloantigen predominantly via the indirect pathway of allorecognition.^6^

Understanding where regulatory T cells function to control rejection at different stages of the immune response after transplantation is critically important, not least for defining assays that can be used to assess whether regulatory T cells are contributing to graft survival in clinical transplantation. In experimental models, the draining lymphoid tissue has been shown to be the primary, initial site of interaction between regulatory T cells and naïve T cells.^25–28^ Later on, regulation also manifests itself within the allograft.^25,28^ Indeed, there are sufficient regulatory T cells present at some graft sites to enable them to control rejection when the graft is transferred to a fresh recipient.^25^ In clinical transplantation, evidence has been obtained that T cells with the phenotypic characteristics of regulatory cells are detectable in both the peripheral blood^29,30^ and within the graft.^31,32^

## References

[1] Sakaguchi S., Sakaguchi N., Asano M. (1995). Immunologic self tolerance maintained by activated T cells expressing IL-2 receptor alpha chains (CD25): Breakdown of a single mechanism of self tolerance causes various autoimmune diseases. J Immunol.

[2] Suri-Payer E., Amar A., Thornton A., Shevach E. (1998). CD4^+^CD25^+^ T cells inhibit both the induction and effector function of autoreactive T cells and represent a unique lineage of immunoregulatory cells. J Immunol.

[3] Baecher-Allan C., Brown J., Freeman G., Hafler D. (2001). CD4^+^CD25^+^ high regulatory cells in human peripheral blood. J Immunol.

[4] Stephens L., Mottet C., Mason D., Powrie F. (2001). Human CD4+CD25+ thymocytes and peripheral T cells have immune suppressive activity in vitro. Eur J Immunol.

[5] Bushell A., Morris P., Wood K. (1995). Transplantation tolerance induced by antigen pretreatment and depleting anti-CD4 antibody depends on CD4^+^ T cell regulation during the induction phase of the response. Eur J Immunol.

[6] Hara M., Kingsley C., Niimi M. (2001). IL-10 is required for regulatory T cells to mediate tolerance to alloantigens in vivo. J Immunol.

[7] Thorstenson K., Khoruts A. (2001). Generation of anergic and potentially immunoregulatory CD25+CD4+ T cells in vivo after induction of peripheral tolerance with intravenous oral antigen. J Immunol.

[8] Kingsley C.I., Karim M., Bushell A.R., Wood K.J. (2002). CD25+CD4+ Regulatory T cells prevent graft rejection: CTLA-4– and IL-10–dependent immunoregulation of alloresponses. J Immunol.

[9] Apostolou I., von Boehmer H. (2004). In vivo instruction of suppressor commitment in naive T cells. J Exp Med.

[10] Cobbold S.P., Castejon R., Adams E. (2004). Induction of foxP3+ regulatory T cells in the periphery of T cell receptor transgenic mice tolerized to transplants. J Immunol.

[11] Marangoni F., Trifari S., Scaramuzza S. (2007). WASP regulates suppressor activity of human and murine CD4+CD25+FOXP3+ natural regulatory T cells. J Exp Med.

[12] Oliveira V., Sawitzki B., Chapman S. (2008). Anti-CD4–mediated selection of Treg in vitro—in vitro suppression does not predict in vivo capacity to prevent graft rejection. Eur J Immunol.

[13] Feng G., Wood K., Bushell A. (2008). Interferon-gamma conditioning ex vivo generates CD25+CD62L+Foxp3+ regulatory T cells that prevent allograft rejection: potential avenues for cellular therapy. Transplanation.

[14] Feng G., Gao W., Strom T.B., Oukka M. (2008). Exogenous IFN-γ ex vivo shapes the alloreactive T-cell repertoire by inhibition of Th17 responses and generation of functional Foxp3+ regulatory T cells. Eur J Immunol.

[15] Francis R.S., Feng G., Tha-In T. (2011). Induction of transplantation tolerance converts potential effector T cells into graft protective regulatory T cells. Eur J Immunol.

[16] Williams L., Rudensky A. (2007). Maintenance of the Foxp3-dependent developmental program in mature regulatory T cells requires continued expression of Foxp3. Nat Immunol.

[17] Hamano K., Rawsthorne M., Bushell A. (1996). Evidence that the continued presence of the organ graft and not peripheral donor microchimerism is essential for the maintenance of tolerance to alloantigen in anti-CD4 treated recipients. Transplantation.

[18] Qin S., Cobbold S.P., Pope H. (1993). “Infectious” transplantation tolerance. Science.

[19] Bushell A., Wood K. (2007). GITR ligation blocks allograft protection by induced CD25+CD4+ regulatory T cells without enhancing effector T-cell function. Am J Transplant.

[20] Warnecke G., Bushell A., Nadig S., Wood K.J. (2007). Regulation of transplant arteriosclerosis by CD25+CD4+ T cells generated to alloantigen in vivo. Transplantation.

[21] Warnecke G., Feng G., Goto R. (2010). CD4+ regulatory T cells generated in vitro with IFN-γ and allogeneic APC inhibit transplant arteriosclerosis. Am J Pathol.

[22] Nadig S.N., Wieckiewicz J., Wu D.C. (2010). In vivo prevention of transplant arteriosclerosis by ex vivo–expanded human regulatory T cells. Nat Med.

[23] Issa F., Hester J., Goto R. (2010). Ex vivo–expanded human regulatory T cells prevent the rejection of skin allografts in a humanised mouse model. Transplantation.

[24] Koshiba T., Kitade T., van Damme B. (2003). Regulatory cell–mediated tolerance does not protect against chronic rejection. Transplantation.

[25] Graca L., Cobbold S.P., Waldmann H. (2002). Identification of regulatory T cells in tolerated allografts. J Exp Med.

[26] Tang Q., Adams J., Tooley A. (2006). Visualizing regulatory T cell control of autoimmune responses in nonobese diabetic mice. Nat Immunol.

[27] Mempel T.R., Pittet M., Khazaie K. (2006). Regulatory T cells reversibly suppress cytotoxic T cell function independent of effector differentiation. Immunity.

[28] Carvalho-Gaspar M., Jones N.D., Luo S. (2008). Location and time-dependent control of rejection by regulatory T cells culminates in a failure to generate memory T cells. J Immunol.

[29] Li Y., Koshiba T., Yoshizawa A. (2004). Analyses of peripheral blood mononuclear cells in operational tolerance after pediatric living donor liver transplantation. Am J Transplant.

[30] Akl A., Jones N.D., Rogers N. (2008). An investigation to assess the potential of CD25highCD4+ T cells to regulate responses to donor alloantigens in clinically stable renal transplant recipients. Transpl Int.

[31] Li Y., Zhao X., Cheng D. (2008). The presence of Foxp3 expressing T cells within grafts of tolerant human liver transplant recipients. Transplantation.

[32] Dijke I.E., Velthuis J.H.L., Caliskan K. (2007). Intragraft FOXP3 mRNA expression reflects antidonor immune reactivity in cardiac allograft patients. Transplantation.

